# Weaning from invasive ventilation with levosimendan (WEANLESS): study protocol for a multicentre randomised clinical trial

**DOI:** 10.1136/bmjopen-2026-117615

**Published:** 2026-05-20

**Authors:** Esther De Leijer, Charlotte Hofma, Ary Serpa Neto, Nicole G M Hunfeld, Marcus J Schultz, Jonne Doorduin, Leo Heunks

**Affiliations:** 1 Department of Intensive Care, Radboudumc, Nijmegen, The Netherlands; 2 Department of Critical Care Medicine, Hospital Israelita Albert Einstein, São Paulo, Brazil; 3 Department of Adult Intensive Care and Hospital Pharmacy, Erasmus MC University Medical Center Rotterdam, Rotterdam, The Netherlands; 4 Department of Intensive Care, Amsterdam UMC Locatie AMC, Amsterdam, The Netherlands

**Keywords:** Adult intensive & critical care, Medicine, Respiratory physiology, Ventilators, Mechanical

## Abstract

**Background:**

Prolonged weaning from invasive mechanical ventilation is a major challenge in critically ill patients failing a spontaneous breathing trial. Levosimendan, a calcium sensitiser, has been shown to improve respiratory muscle function. However, its effect on clinically relevant endpoints in difficult to wean patients has not yet been investigated. We aim to assess whether levosimendan shortens the weaning process in invasively ventilated intensive care unit (ICU) patients who fail a separation attempt. The objective is to assess the effect of levosimendan on the number of ventilator-free days and alive at day 28.

**Methods:**

The WEANing with LEvoSimendan Study (WEANLESS) is an investigator-initiated, multicentre, double-blind, parallel-group, randomised clinical superiority trial. Adult invasively ventilated patients who failed a separation attempt are randomly assigned to receive either intravenous levosimendan (intervention) or placebo (control). The primary outcome is the number of ventilator-free days and alive at day 28 from randomisation. WEANLESS also evaluates the effects of levosimendan on patient-reported outcomes, measured through daily dyspnoea scores and uses an EQ-5D-5L questionnaire. Additionally, we will evaluate healthcare resource utilisation and intensive care capacity, assessed through reintubation rates, ICU readmissions within 90 days, the need for non-invasive respiratory support and ICU length of stay. WEANLESS includes a pharmacokinetic analysis of levosimendan and its metabolites.

**Discussion:**

WEANLESS is the first clinical study that is sufficiently powered to determine the effect of intravenous levosimendan in difficult to wean patients on the duration of weaning from invasive ventilation.

**Ethics and dissemination:**

WEANLESS is registered before the inclusion of the first patient at clinicaltrials.gov; the study protocol has been approved by the relevant ethics committee. Its findings will be disseminated through presentations at scientific conferences and publications in a peer-reviewed journal.

**Trial registration number:**

NCT07105202.

**STRENGTHS AND LIMITATIONS OF THIS STUDY**

- Use of a clinically meaningful and well-defined primary endpoint: ventilator free days and alive (VFD) up to day 28, this composite measure captures both liberation from ventilation and survival, reflecting the goals of intensive care unit (ICU) care.
- With VFD as a composite endpoint, it is not possible to distinguish between patients who die early (who automatically receive a score of 0) and patients who are never successfully extubated within 28 days.
- This study has a multicentre, double-blind design, which enhances both internal validity and external generalisability across diverse ICU settings.

## Introduction

### Background

 Acute respiratory failure is a life-threatening condition that may develop in the course of many diseases, including pneumonia, sepsis, trauma, chronic lung diseases or after surgery. Especially the elderly, patients with comorbidities and immunocompromised patients are at risk of developing acute respiratory failure. In these patients, invasive ventilation is a lifesaving intervention. However, invasive ventilation is a double-edged sword. While being lifesaving, invasive ventilation is associated with serious complications which are directly related to the duration of the ventilation. Successful liberation of patients from invasive ventilation (‘weaning’), represents a crucial step in the recovery process following severe lung failure. Unfortunately weaning can be a complicated process. A prolonged weaning duration worsens patient outcomes, increases the risk of death and extends the length of stay in the intensive care unit (ICU).^1^ Duration of invasive ventilation is among the strongest predictors of 1 year functional outcome.^2^ In addition, weaning duration impacts healthcare resource utilisation. Costs of ICU are very high, in the Netherlands ±3000 euro per day and critical care resources are limited. Therefore, reducing duration of ventilator weaning affects direct financial healthcare costs and the opportunity for other patients of consumption of finite critical care capacity. In conclusion, early ventilator weaning is important for the benefit of the individual patient, but also for societal benefit.

ICU acquired weakness of the respiratory muscles plays a significant role in weaning from invasive ventilation.^3^ A disbalance in the load imposed on the respiratory muscles and capacity of the respiratory muscles results in ventilator weaning failure. In patients ventilated >24 hours, 63% developed respiratory muscle weakness at the time of weaning.^3^ No medical intervention has been shown effective in improving respiratory muscle function in ICU patients. This is surprising given that respiratory muscle weakness frequently develops in ventilated patients and is associated with adverse outcome. Therefore, interventions that improve respiratory muscle function in patients difficult to wean from invasive ventilation are much needed. The calcium sensitiser levosimendan has shown positive effects on the respiratory muscles in experimental models, healthy subjects and ventilated ICU patients.^4^,^6^ The effects on weaning outcome are unknown.

### Clinical trial rationale

Respiratory muscle weakness frequently develops in invasively ventilated patients and is strongly associated with adverse clinical outcome.^3 7^ Respiratory muscle weakness in ventilated patients is partly explained by muscle atrophy,^8^ but also by impaired force generation at a given intracellular calcium concentration, that is, a so-called reduced calcium sensitivity of contraction.^9^ Reduced calcium sensitivity reduces contractile efficiency, that is, more energy is needed and more CO_2_ is generated as compared with non-ICU patients. Levosimendan is a cardiac inotrope that improves calcium sensitivity of force generation of cardiac muscle and is approved for treatment of acute heart failure. Regarding the diaphragm, research followed a stepwise approach to investigate the effects of levosimendan on diaphragm function, progressively moving from basic to clinical studies. First, levosimendan improved the calcium sensitivity of force generation in diaphragm fibres.^4^ This was the very first study to demonstrate that pharmacological modulation of the troponin complex improves human diaphragm function. Second, in a double-blind placebo-controlled trial in healthy subjects, levosimendan improved in vivo contractile efficiency of the diaphragm by 21%.^5^ Levosimendan also restored diaphragm contractility after loaded breathing (reversal of muscle fatigue). Third, in a double-blind placebo-controlled study, we demonstrated that levosimendan increased tidal volume, minute ventilation and decreased arterial CO_2_ in critically ill ventilated patients.^6^ No serious adverse events occurred. In summary, previous work demonstrated that respiratory muscles of invasively ventilated patients show impaired calcium sensitivity of contraction and that levosimendan improves calcium sensitivity. Given these results, we hypothesise that levosimendan shortens the weaning process by enhancing respiratory muscle function, ultimately leading to a reduced ICU length of stay and lower mortality rates compared with standard care.

### Objectives

The primary objective of WEANing with LEvoSimendan Study (WEANLESS) is to determine the effect of intravenous levosimendan on the number of ventilator free days and alive (VFD) at day 28 after randomisation. This is a composite endpoint combining both mortality and the duration of ventilation. A patient who dies before day 28 will be assigned 0 VFD. Day 0 will be defined as the day of randomisation.

Secondary objectives include evaluating the effects of levosimendan on patient-reported outcomes, measured through daily dyspnoea scores and quality of life measured with the the EuroQol five-dimensional (EQ-5D-5L) questionnaire. The study will also investigate its impact on healthcare resource utilisation and intensive care capacity, assessed through reintubation rates, ICU readmission within 90 days, the need for non-invasive respiratory support, ICU length of stay and mortality. Furthermore, the pharmacokinetics of levosimendan and its metabolites will be analysed in a subset of study patients, with measurements taken up to 120 hours after infusion of the medication.

### Trial design

The WEANLESS is an investigator-initiated, multicentre, double-blind, parallel-group, randomised clinical superiority trial with a 1:1 allocation ratio. Participants will be randomised to receive either intravenous levosimendan or placebo. The study is registered in a publicly accessible clinical trial database.

## Methods: participants, interventions and outcomes

### Patient and public involvement

Before drafting the protocol, consultations were held with patient representatives from the Netherlands Patients Federation, Lung Foundation Netherlands and FCIC. Invasively ventilated patients often describe dyspnoea as one of the most distressing experiences in the ICU.^10^ It is a major contributor to ICU-related post-traumatic stress disorder and reduced quality of life.^11 12^ Given its significant impact, dyspnoea has been identified as a key secondary endpoint in this trial.

### Study setting

The trial will be conducted in the ICUs of participating (academic and top-clinical) hospitals in the Netherlands. A current list of participating centres can be found on https://clinicaltrials.gov/.

#### Population

WEANLESS will include patients aged over 18 years, admitted to participating ICU, ventilated for more than 48 hours and who have at least one failed separation attempt, defined as a failed spontaneous breathing trial (SBT, see SBT failure criteria) or a failed extubation (reintubation within 7 days) (‘difficult to wean from invasive ventilation’).

### Eligibility criteria

**Figure 1:**
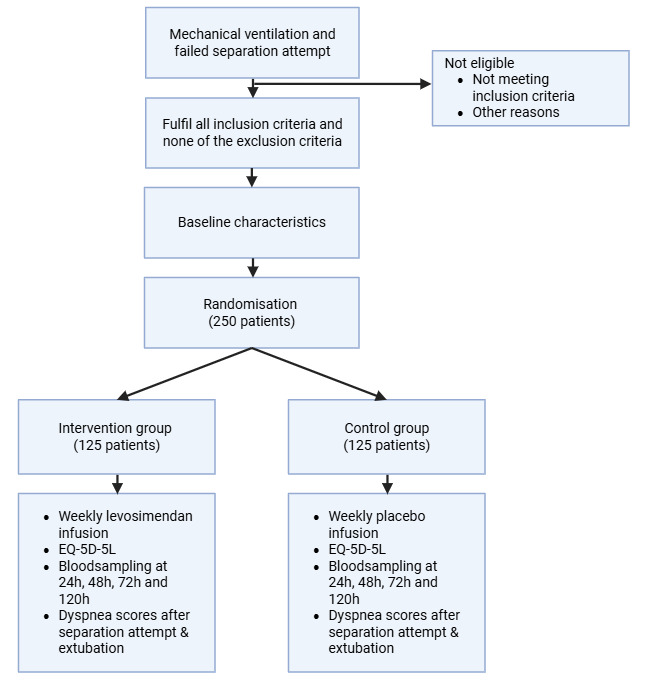
Flow diagram for enrolment of a WEANLESS participant.

Patients are eligible for participation in WEANLESS if: (1) admitted to a participating ICU; (2) aged 18 years or older; (3) invasively ventilated for more than 48 hours and (4) have at least one failed separation attempt (a failed SBT or failed extubation). The following exclusion criteria have been defined: pre-existing neuromuscular disease, endotracheally intubated primarily for neurological reason, treatment with intermittent haemodialysis, treatment limitation decision in place: do not reintubate, previous treatment with levosimendan within 30 days, currently in another interventional trial or contraindications for levosimendan. Contraindications for levosimendan are: severe renal failure (creatinine clearance <30 mL/min) unless managed with continuous appropriate kidney replacement therapy (such as continuous veno-venous haemofiltration (CVVH)), severe liver failure (Child-Pugh class C), history of torsade des pointes; known significant mechanical obstructions affecting ventricular filling/outflow or both; prolonged QTc interval (QTc >470 ms); pregnancy, breast feeding; known hypersensitivity to levosimendan. See figure 1 for the enrolment procedure.

### Informed consent

If a patient is deemed potentially eligible, a member of the study team may approach the patient or their proxy even before the patient undergoes a separation attempt to provide information and obtain consent. Only if the patient subsequently fails the separation attempt, the study will proceed with randomisation and administration of the study medication. In cases where the patient or proxy has not yet been informed at the time of the failed separation attempt, the study team will initiate the informed consent process as soon as possible after separation failure. A window of up to 24 hours after a failed separation attempt is allowed to obtain informed consent. Given the critical condition of ICU patients, deferred consent procedures will frequently be applied by obtaining consent from a legal representative. If and when the patient regains decision-making capacity, informed consent will be asked from the patient. The patient information form is provided in the supplementary material.

### Additional consent provisions for collection and use of participant data and biological specimens

Separate consent will be obtained for the collection, storage and future use of data and biological samples for research beyond the primary study objectives. This will be addressed in a dedicated section of the consent form.

### Interventions

#### Explanation for the choice of comparators

Patients who meet all the inclusion criteria and none of the exclusion criteria will be randomised to receive either the intervention (intravenous levosimendan) or placebo, in addition to standard care for weaning from mechanical ventilation. A placebo was selected as comparator because no active comparator exists for this indication.

The placebo group, like the intervention group, will receive standard ICU care for weaning from mechanical ventilation. To ensure comparability between groups and uniformity across study sites, the weaning process will be standardised using predefined criteria for initiating and assessing SBTs and extubation.

#### Weaning process

To standardise the weaning process across all study sites, physicians are required to assess daily whether patients meet the criteria for an SBT. The criteria align with the weaning protocol developed by the Dutch Society of Intensive Care,^13^ which is supported by recent literature.^14^,^16^ We strongly encourage physicians to adhere to the criteria outlined below.

Criteria for initiating an SBT:

1. Adequate oxygenation: oxygen saturation (SpO_2_) >90% with fraction of inspired oxygen (FiO_2_) ≤50% and positive end expiratory pressure (PEEP) ≤8 cmH_2_O.
2. Adequate respiratory rate: respiratory rate ≤34/min.
3. Stable cardiovascular status: systolic blood pressure 90–160 mm Hg without or with minimal vasopressor use, heart rate ≤140/min.

When a patient meets all of the above criteria, an SBT will be performed. This trial should last at least 30 min and can be conducted using one of the following methods:

1. Using the T-piece.
2. Pressure support mode, with pressure support (PS) ≤8 cmH_2_O and PEEP ≤7 cmH_2_O.
3. Continuous positive airway pressure (CPAP) mode, with PEEP ≤7 cmH_2_O.

#### SBT failure criteria

SBT failure will be documented in the electronic case report form (eCRF) if the patient develops any of the following:

- Worsening oxygenation (SpO_2_ <90% with FiO_2_ ≥50%).
- Acute distress (respiratory rate≥40 /min, agitation, cyanosis).
- Haemodynamic instability (systolic blood pressure ≤90 or ≥180 mm Hg, heart rate >140 /min or increase of 20%.
- Change in mental status.

#### Extubation

Following a successful SBT, extubation should be considered eligible for extubation if the following conditions are met:

- Completed a successful SBT (patient did not meet any of the failure criteria listed above).
- Cooperative cognitive state, as determined by the treating physician.
- Adequate cough, as determined by the treating physician.
- Frequency of suctioning is greater than every 2 hours.

The decision to extubate remains however at the discretion of the treating intensivist. Prophylactic intervention, including non-invasive ventilation, high flow nasal cannula, or both, should be considered for patients meeting any of the following criteria:

- ≥65 years of age.
- Underlying cardiac disease.
- Underlying respiratory disease.

The study protocol does not mandate specific reintubation criteria but requires documentation of all reintubation events.

### Intervention description

#### Levosimendan

After randomisation, subjects will receive either levosimendan or a placebo at a starting dose of 0.1 µg/kg/min,^17^,^19^ diluted with glucose 5%. Use of an initial bolus of levosimendan is generally not recommended, mostly to minimise the risk of vasodilation.^20^ In absence of rate limiting effects (heart rate >130/min, hypotension defined as mean arterial blood pressure (MAP) <65 mm Hg, infusion rate is increased after 4 hours to 0.2 µg/kg/min for the remaining 20 hours. If the patient has rate-limiting side effects at the dose of 0.2 µg/kg/min infusion rate is reduced to 0.1 µg/kg/min or less or even discontinued.^6 21^ This dosage will be repeated weekly until successful weaning from invasive ventilator or until the primary endpoint is reached, with a maximum of four doses administered.

#### Placebo

The placebo group will receive intravenous multivitamins (Soluvit N) as a placebo. When diluted (10 mL in 500 mL glucose 5%), Soluvit N provides a yellow colour that matches the characteristic appearance of levosimendan. This is consistent with practices in other intervention trials with levosimendan,^22^,^25^ LevoHeartShock (NCT04020263). The route, volume and infusion rate are matched with the administration of levosimendan to maintain blinding during the study.

### Criteria for discontinuing or modifying allocated interventions

Study medication (levosimendan or placebo) will be administered intravenously at a starting rate of 0.1 µg/kg/min, with the option to increase to 0.2 µg/kg/min after 4 hours if well tolerated. If a patient develops haemodynamic instability, defined as a heart rate >130 beats/min or hypotension with mean arterial pressure <65 mm Hg, the infusion rate will be reduced to 0.1 µg/kg/min or lower, or discontinued altogether if necessary or if requested by the patient. Any discontinuation of treatment will be documented in the electronic case report form, including the underlying reason.

### Strategies to improve adherence to interventions

To ensure adherence to the study protocol, each study site will have a dedicated team trained by the steering committee. Training will include an initiation visit, review of standard operating procedures (SOPs), and a clinical lesson for all nurses and physicians involved in the trial. Additionally, the research pharmacy will receive an SOP for proper preparation and blinding of study medications. ICU nurses will also receive an SOP on infusion rates and when to adjust dosages, with close monitoring of both adjustments and side effects.

The weaning process will be standardised as much as possible, with daily assessments by the intensivist to determine whether SBT criteria are met.

Any deviations from the study protocol will be documented in the eCRF and evaluated during data safety monitoring board (DSMB) meetings.

### Relevant concomitant care permitted or prohibited during the trial

Treating physicians may prescribe any concomitant medications or treatments deemed necessary to provide adequate patient care without restrictions due to this research. Vasopressors, inotropes and antiarrhythmic medications will be considered relevant medications and must be recorded in the eCRF.

### Provisions for post-trial care

Not applicable. There is no need for provisions for post-trial care after the patient is discharged from the ICU.

### Outcomes

The primary endpoint of the study is the number of ventilator-free days and alive (VFD) at day 28 from randomisation. This is a composite endpoint combining both mortality and the duration of ventilation. In case of death, the subject will be assigned a value of 0 VFD. Day 0 will be defined as the day of randomisation.

Secondary outcomes include ICU mortality, VFD from randomisation up until day 90, safety endpoints such as hypotension (MAP <65 mm Hg), cardiac dysrhythmia (atrial fibrillation, ventricular arrhythmias), dyspnoea sensation, EQ-5D-5L questionnaire at baseline, 3 months and 12 months, reintubation rate, non-invasive support after detubation and pharmacokinetics of levosimendan up until 5 days.

### Participant timeline

The time points for enrollment, interventions and follow-up can be found in table 1.

**Table 1:** Participant timeline

	Pretreatment						Follow-up
	Day −1	Day 0	Day 1	Day 2, 3, 4, 6	Day 8, 15, 22	Day 28	Day 90, 365
Enrolment							
Eligibility screening	X						
Baseline characteristics		X					
Informed consent*		X					
Randomisation		X					
Interventions							
EQ-5D-5L		X					X
Study medication†			X		X		
Baseline lab‡		X					
TDM levosimendan				X			
Dyspnoea scores			Daily from extubation up until day 28/ICU discharge				
Assessments							
Ventilation data			Daily				
Adverse events/safety endpoints			Daily				
Reintubation rate			Daily from extubation up until day 28/ICU discharge				
Life status, duration of ventilation, ICU and hospital admission						X	X

* Informed consent is obtained before randomisation.

† Patients will receive the study medication weekly until successful weaning or until the primary endpoint is reached, with a maximum of four doses administered.

‡ In baseline lab we will include NTproBNP as a marker of cardiac failure and in case of a female a pregnancy test.

ICU, intensive care unit; NT-proBNP, N-terminal pro b-type natriuretic peptide; TDM, therapeutic drug monitoring.

### Sample size

The mean and SD of ventilator-free days at day 28 (VFD-28) are not precisely known for our target population (patients who are invasively ventilated and have a failed separation attempt). Based on data from the PReVENT dataset,^26^ we estimate the mean VFD-28 to be 15.6 days, with an expected SD of 10.6.

We aim to detect a clinically meaningful improvement of 25% in VFD-28, corresponding to an absolute difference of 3.9 days. A sample size of 117 patients per group provides 80% power to detect this difference using a two-sided alpha level of 0.05. To account for potential non-normality in the distribution of VFD-28, we have increased the sample size to 125 patients per group, resulting in a total sample size of 250 patients.

### Recruitment

With a sample size of 250 patients and an inclusion rate of 10 patients/months we expect that the recruitment period is approximately 2 years after all eight centres start enrolling patients. Eligible patients will be identified from ICUs in participating hospitals where patients are receiving invasive ventilation based on daily screening on the study’s inclusion and exclusion criteria.

### Assignment of interventions: allocation

#### Sequence generation

Clinical research platform Castor Electronic Data Capture (https://www.castoredc.com/) will be used to perform the randomisation and is Good Clinical Practice. Participants will be randomised in a 1:1 allocation ratio to ensure equal distribution between the intervention and control groups. To minimise potential site-specific effects, stratified randomisation by study centre will be applied.

#### Concealment mechanism

Patients will be randomised in Castor after obtaining informed consent. Concealment will be ensured by the use of blocks with randomly permuted sizes.

#### Implementation

The local principal investigator, who is trained by the steering committee, and the dedicated study team on the study site will enrol and randomise patients and make sure they will receive the dedicated strategy. The unblinded team, primarily pharmacy personnel, will receive the randomisation results and ensure that the assigned medication is provided to the nurse for administration to the patient.

### Assignment of interventions: blinding

#### Who will be blinded

This study will follow a double-blind design, meaning that both participants and all investigators involved in recruitment and outcome assessment will remain blinded to treatment assignments at all times.

#### Procedure for unblinding if needed

Emergency unblinding, however, remains possible. This is strictly limited to situations where knowing the patient’s assigned treatment is critical for safety, such as life-treating arrhythmias or severe adverse events. In such cases, the investigator will contact the unblinded team, who will provide the information about the treatment assignment.

### Data collection and management

#### Plans for assessment and collection of outcomes

Most study data will be collected from electronic patient records, ensuring accurate and efficient documentation. Baseline characteristics will be recorded on enrolment, including demographic data, medical history, Acute Physiology And Chronic Health Evaluation II (APACHE II) and Sequential Organ Failure Assessment (SOFA) scores, ECG, medication use, intoxication and baseline laboratory. If a cardiac echocardiogram is performed as part of standard care to evaluate potential weaning failure,^27^ data on ejection fraction and diastolic function will be collected. However, if a cardiac ultrasound is not performed as part of standard care, no additional ultrasound will be conducted for study purposes.

Clinical parameters including ventilator settings, respiratory mechanics, haemodynamic status and laboratory values will be recorded daily until day 28 or ICU discharge. Blood samples will be collected in selected centres to assess the pharmacokinetics of levosimendan in critically ill patients. Baseline NT-proBNP measurements will be used to identify patients with a cardiac origin of weaning failure. Pharmacokinetic assessments will be conducted at 24, 48, 72 and 120 hours after completion of the first dose of the study medication. While blood sampling will be performed in most patients, logistical constraints may prevent its feasibility in all participating hospitals, meaning not all patients will undergo these assessments.

Dyspnoea scores will be assessed using a standardised daily dyspnoea scale. The score will only be evaluated in patients with a Richmond Agitation-Sedation Scale score between −2 and +2, and in the absence of delirium or confusion as determined by the confusion assessment method for the intensive care unit (CAM-ICU) assessment. After confirming adequate mental status using two trigger questions, patients will be asked whether they are experiencing dyspnoea. If they confirm the presence of dyspnoea, a visual analogue scale (VAS) ranging from 0 to 10 will be used to quantify its severity. The EuroQol 5-dimensions 5-level questionnaire is a standardised instrument to describe and value health, consisting of a descriptive system and a VAS. The EQ-5D-5L will be performed at baseline (describing the situation before ICU admission), after 3 months and after 12 months.

Healthcare resource utilisation including reintubation rates, ICU readmission within 90 days, need for non-invasive respiratory support, mortality and ICU length of stay will be retrieved from electronic patient records and documented in the eCRF to perform a Health Technology Assessment analysis.

#### Plans to promote participant retention and complete follow-up

Almost all relevant study data in the WEANLESS study can be extracted from the patient’s medical record, minimising the risk of loss to follow-up for the primary endpoint. Since all participants are admitted to the ICU, missing data is expected to be minimal. To further reduce the risk of loss to follow-up, patients and relatives are informed about the follow-up process, the informed consent procedure and the research team’s contact details are provided on all consent forms.

### Data management

To maintain data integrity, research personnel at each site will be trained in standardised data collection procedures, automated validation checks within the eCRF will flag inconsistencies or missing data for review and data entry will include range checks and automated calculations to minimise errors. All study data will be securely stored and retained for 15 years after study completion, following regulatory guidelines. Blood samples will be processed and stored according to study protocol. No biological specimens will be used for genetic or molecular research, either within this trial or for future studies.

### Confidentiality

Patient data will be pseudonymised using a unique identification code that is not linked to personal identifiers. All personal data handling will comply with the General Data Protection Regulation. Coded study data may be shared between participating hospitals using secure data-sharing platforms designed for clinical research.

### Plans for collection, laboratory evaluation and storage of biological specimens for genetic or molecular analysis in this trial/future use

Blood samples will be collected for analysis of pharmacokinetics and future research. It will be stored for a maximum of 10 years in the Radboudumc. Patients may provide separate consent for the use and storage of the biological specimens.

### Statistical methods

#### Statistical methods for primary and secondary outcomes

For data analysis, an intention-to-treat analysis will be performed on the primary outcome, with patients analysed according to their assigned treatment arms, except for cases lost to follow-up, or patients who are withdrawn due to lack of deferred informed consent. Statistical uncertainty will be expressed by the 95% confidence levels with p value under 0.05 will be considered statistically significant.

The primary analysis of the primary outcome, which is ventilator-free days up until day 28 (VFD-28), will reflect a composite endpoint combining both mortality and the duration of ventilation. This approach integrates the impact of death (assigned a value of 0 VFD) and the length of time a patient remains on invasive ventilation. Day 0 will be defined as the day of randomisation, with the duration of intubation prior to randomisation reported as a baseline variable. The individual components of the composite endpoint, mortality rates and the duration of ventilation, will be described separately for both treatment groups to provide detailed insights into the effects of the intervention. The primary analysis for VFD-28 will use a cumulative logistic regression model to account for the ordinal nature of the endpoint. This model will estimate ORs and 95% CIs to quantify the effect of the treatment compared with the control group.

A pre-planned secondary analysis will be conducted focusing on patients with cardiac failure. This analysis will specifically assess this subgroup to explore potential differential effects of treatment. Patients with cardiac failure will be identified using a baseline NT-proBNP level.

A full statistical analysis plan will be made available before the closing of the database.

#### Interim analyses

No interim analysis for efficacy will be performed during this study. An independent DSMB will assess the safety of patients throughout the study.

#### Methods for additional analysis (eg, subgroup analyses)

A pre-planned secondary analysis will be conducted focusing on patients with cardiac failure as primary reason for intubation.

#### Methods in analysis to handle protocol non-adherence and any statistical methods to handle missing data

Protocol deviations will be tracked in the eCRF. If protocol adherence is found to be insufficient, the DSMB will assess the impact on study integrity and may recommend corrective measures, such as additional training for site personnel, protocol clarifications or increased monitoring at participating centres. Since nearly all required study data is available from patient records, the proportion of missing data is expected to be minimal. If data for the primary endpoint is missing, a complete case analysis will be conducted. For all other variables, multiple imputation will be the primary method used to address missing data.

#### Plans to give access to the full protocol, participant level-data and statistical code

In accordance with the FAIR (findable, accessible, interoperable and reusable) data principles, study data will be managed and shared in a way that promotes transparency, enables reuse and facilitates future research, while safeguarding participant confidentiality. The study protocol is publicly available, and after study completion, anonymised participant-level data and statistical code will be accessible on reasonable request, in compliance with ethical guidelines and data protection regulations.

### Oversight and monitoring

#### Composition of the coordinating center and trial steering committee

The coordinating centre at Radboudumc (Nijmegen, the Netherlands) is responsible for the overall management of the trial, including coordination between sites, ensuring regulatory compliance and maintaining communication with all participating centres. The coordinating centres also oversee data management, safety reporting and protocol adherence across all sites.

The steering committee of the WEANLESS study consists of five researchers including the principal investigator, with mostly different fields of expertise and with different responsibilities. They will meet every other week and review study progress, discuss operational challenges and discuss necessary protocol amendments.

Each participating centre has a designated local investigator responsible for the day-to-day conduct of the trial at their site. This includes participant screening and enrolment, informed consent procedures, data collection, adherence to the study protocol and timely reporting of adverse events.

#### Composition of the data monitoring committee, its role and reporting structure

A DSMB will be established to oversee the safety of the study interventions and the overall conduct of the trial. The DSMB will consist of four independent experts who are not involved in the conduct of the trial. Their primary responsibilities will include safeguarding patient safety and monitoring the progress and integrity of the study.

Beyond evaluating participant safety, the DSMB will review key aspects of trial execution, such as recruitment rates, protocol adherence and any emerging challenges that could impact study validity or feasibility. This includes assessing enrolment progress, identifying frequent protocol deviations and addressing operational challenges as needed.

The DSMB will convene at predefined milestones: after the inclusion of 25 patients, at 50% and 75% of total enrolment, or at least within 9 months after the first patient is enrolled. During these meetings, they will assess trial data and provide recommendations to ensure the study remains on course.

The DSMB’s recommendations will be communicated exclusively to the study sponsor. If the sponsor chooses not to fully implement a DSMB recommendation, they must submit the advice, along with a justification, to the reviewing medical ethics review board (METC).

### Adverse event reporting and harms

To ensure patient safety, participants will be closely monitored during the administration of the study medication. Any adverse events occurring during the intervention period will be documented.

### Frequency and plans for auditing trial conduct

Monitoring will be carried out by an independent monitor. The stored data in the CRFs, informed consents, serious adverse events (SAE) reports and the Trial Master File will be monitored, as described in the approved monitoring plan. In the monitor plan we classify our study as a ‘minimal risk study’.

### Plans for communicating important protocol amendments to relevant parties (trial participants, ethical committees)

Every substantial amendment must be reported and approved by the ethical board of the sponsor. Every participating centre and other relevant parties will be notified of the approval of an amendment by email and trial registries will be updated.

## Dissemination plans

The results arising from this randomised controlled trial (RCT) will be presented at scientific meetings as abstracts for poster or oral presentations and published in peer-reviewed journals. Participants can find the results of the study on https://clinicaltrials.gov/ under the name WEANLESS or with the identifier NCT07105202.

## Ethics

The sponsor has an insurance that is in accordance with the legal requirements in the Netherlands (Article 7 WMO, under 1). This insurance provides cover for damage to research subjects through injury or death caused by the study. The insurance applies to the damage that becomes apparent during the study or within 4 years after the end of the study.

- €650 000—for death or injury for each subject who participates in the research.
- €5 000 000—for death or injury for all subjects who participate in the research.
- €7 500 000—for the total damage incurred by the organisation for all damage disclosed by scientific research for the Sponsor as ‘verrichter’ in the meaning of said Act in each year of insurance coverage.

## Discussion

Prolonged weaning from invasive ventilation is a major challenge in critically ill patients, contributing to increased morbidity, mortality and healthcare burden. The WEANLESS study is the first randomised controlled trial to evaluate whether intravenous levosimendan can increase the number of ventilator free days from randomisation to day 28 in ICU patients.

Several studies have suggested that levosimendan, a calcium sensitiser and vasodilator, may improve diaphragm contractility and enhance respiratory muscle function,^5^ thereby facilitating weaning in patients with difficult-to-wean conditions. Previous trials in cardiac surgery and sepsis-related myocardial dysfunction have demonstrated its potential benefits and safety in critically ill patients, including improved cardiac output, reduced need for vasopressors and enhanced oxygen delivery to tissues.^28^ However, its specific role in the weaning process from mechanical ventilation remains uncertain.

A notable strength of this study is the use of a clinically meaningful and well-defined primary endpoint: VFD up to day 28. This composite measure captures both liberation from ventilation and survival, reflecting the goals of ICU care. Importantly, our study defines day 0 as the moment of randomisation, not the start of mechanical ventilation, which more accurately reflects the start of the weaning intervention. Nonetheless, VFD as a composite endpoint has its limitations. For example, it is not possible to distinguish between patients who die early (who automatically receive a score of 0) and patients who are never successfully extubated within 28 days. Therefore, these distinctions will be addressed through secondary outcomes such as reintubation rates and ICU length of stay.

Another strength of the WEANLESS study is its multicentre, double-blind design, which enhances both internal validity and external generalisability across diverse ICU settings. Standardised protocols for SBTs and weaning criteria further reduce centre-level variation and potential bias, even though blinding of the weaning process itself is not feasible due to the need for active physician management. The intervention, however, remains blinded to both treating clinicians and outcome assessors.

Given the heterogeneity of ICU patients, we prespecified patient characteristics of interest (eg, presence of cardiac dysfunction) that may influence response to levosimendan. While the study is powered to detect an overall effect of the intervention on VFDs, it may lack sufficient power to detect differences within smaller subgroups. Therefore, we will also perform interaction analyses to assess whether the effect of the intervention differs across these prespecified subgroups, providing a more robust and interpretable evaluation of potential effect modification.

In conclusion, the WEANLESS study is designed to provide valuable insights into the role of levosimendan in weaning from invasive ventilation. If found effective in reducing weaning time and improving clinical outcomes, this study could pave the way for the first pharmacological approach to support the weaning process in critically ill patients.

### Trial status

The WEANLESS study (protocol version 4, December 2025) is scheduled to begin recruitment in September 2025. Recruitment is expected to be completed by September 2027.
